# Seven years of the field epidemiology training programme (FETP) at Chennai, Tamil Nadu, India: an internal evaluation

**DOI:** 10.1186/1478-4491-10-36

**Published:** 2012-09-27

**Authors:** Tarun Bhatnagar, Mohan D Gupte, Yvan J Hutin, Prabhdeep Kaur, Vasanthapuram Kumaraswami, Ponnaiah Manickam, Manoj Murhekar, Vidya Ramachandran, Ramachandran Ramakrishnan

**Affiliations:** 1 National Institute of Epidemiology (NIE), Indian Council of Medical Research (ICMR), Chennai, TN, 600077, India; 2 WHO India country office, New Delhi, India

## Abstract

**Background:**

During 2001–2007, the National Institute of Epidemiology (NIE), Chennai, Tamil Nadu, India admitted 80 trainees in its two-year Field Epidemiology Training Programme (FETP). We evaluated the first seven years of the programme to identify strengths and weaknesses.

**Methods:**

We identified core components of the programme and broke them down into input, process, output and outcome. We developed critical indicators to reflect the logic model. We reviewed documents including fieldwork reports, abstracts listed in proceedings and papers published in Medline-indexed journals. We conducted an anonymous online survey of the graduates to collect information on self-perceived competencies, learning activities, field assignments, supervision, curriculum, relevance to career goals, strengths and weaknesses.

**Results:**

Of the 80 students recruited during 2001–2007, 69 (86%) acquired seven core competencies (epidemiology, surveillance, outbreaks, research, human subjects protection, communication and management) and graduated through completion of at least six field assignments. The faculty-to-student ratio ranged between 0.4 and 0.12 (expected: 0.25). The curriculum was continuously adapted with all resources available on-line. Fieldwork led to the production of 158 scientific communications presented at international meetings and to 29 manuscripts accepted in indexed, peer-reviewed journals. The online survey showed that while most graduates acquired competencies, unmet needs persisted in laboratory sciences, data analysis tools and faculty-to-student ratio.

**Conclusions:**

NIE adapted the international FETP model to India. However, further efforts are required to scale up the programme and to develop career tracks for field epidemiologists in the country.

## Introduction

In 1951, the United States of America Centers for Disease Control and prevention (CDC) launched the Epidemic Intelligence Service (EIS) to recruit young professionals and train them in outbreak investigations and applied epidemiology [1]. Fifty years later, the programme had become a reference [2,3]. Later on, similar programmes started around the world under the name Field Epidemiology Training Programmes (FETP) and Public Health Schools without Walls (PHSWW) [4,5]. FETPs retained the key elements of EIS in terms of learning-through-service during a two-year fellowship but also adapted themselves to fit into the national environments. International FETPs are now networked worldwide through the Training in Epidemiology and Public Health Interventions NETwork (TEPHINET) [6].

Few published articles are available that report evaluations of FETPs [7-9]. Evaluations may have been conducted at the request of various stakeholders or donors, but they have not been made available in the public domain through publications in peer-review journals.

In 2001, the National Institute of Epidemiology (NIE) under the Indian Council of Medical Research (ICMR) started a national, two-year FETP with its base in Chennai, Tamil Nadu, with initial support of the World Health Organization (WHO), CDC and the Australian FETP. In 2009, we decided to apply the principles we teach to our own activities and evaluated our FETP internally using a standard programme evaluation framework [10]. Our objective was to identify the strengths and weaknesses of the programme so that we could improve it.

## Methods

### Overall approach

We described the programme through internal discussions and review of documents. We identified the core components of the programme and broke them down into input, process, output and outcome. We developed a limited set of critical indicators to reflect the logic model.

### Data collection

We collected information to document the indicators. First, we reviewed the programme documentation and records. Second, we reviewed fieldwork reports and final research project reports. Third, we reviewed abstracts listed in conference proceedings and papers accepted in Medline-indexed journals. Fourth, we surveyed the graduates. We used the graduates' e-mail forum to circulate a questionnaire with a majority of close-ended questions and three general open-ended questions for anonymous self-administration using an Internet-based survey tool (http://www.surveymonkey.com). The questionnaire addressed: (1) self-perceived competencies before and after FETP, (2) learning activities, (3) field assignments, (4) supervision, (5) curriculum, (6) relevance to career goals, (7) strengths, and (8) weaknesses. We informed graduates of the purpose of the survey, obtained on-line approval to participate and did not keep track of any direct (e.g., name) or indirect (e.g., age, sex, Internet protocol address) identifier. This evaluation of training activities was exempt from ethical committee review.

### Data analysis

We captured the whole population of graduates and calculated indicators in proportions expressed in percentages.

## Results

### Programme description

The programme was a variant of the international FETP model that built on the learning-through-service EIS model and the academic roots of the PHSWWs. NIE is one of the institutes under ICMR, the autonomous medical research arm of the Department of Health Research of the Indian Ministry of Health and Family Welfare. The programme admitted graduates of Bachelor of Medicine and Bachelor of Surgery (MBBS) with at least three years of experience, most often nominated and sponsored by the states. Students, called “scholars”, entered the programme with a three-month induction course at NIE. This was followed by three six-month field postings in their district. There, they worked within the public health service, but as an epidemiologist-in-training, relieved of their former duties. After each of the first and the second field postings, students returned to NIE for a one-month contact session. After the third and last field posting, students came for a last one-month contact session to deliver their final reports.

The contact sessions at NIE consisted of theoretical classes, problem-solving case studies and practical exercises. From 2005 onward, the induction course included a field exercise consisting of a real investigation. Some of these field exercises resulted in publications [11-13]. From the second contact session onwards, they included peer-review sessions in which students received comments about their field project reports or protocols.

Each field posting was associated with the expected submission of deliverable(s). These included a situation analysis, a surveillance data analysis, a surveillance system evaluation, two critiques of scientific articles, a public health programme evaluation and an operational research project. During each of the three field postings, students were supposed to receive at least one supportive supervision visit from a NIE-based mentor. These supervision visits were either in the form of one-to-one student-mentor sessions or an on-site mini-contact session for states with more than one student. Finally, students had to investigate at least one outbreak of disease in two years.

Upon completion, students received a Master of Applied Epidemiology (MAE) degree from the Sree Chitra Tirunal Institute for Medical Sciences and Technology (SCTIMST), Thiruvanathapuram, Kerala, India. After graduation, they returned to their parent state and worked in public health. The institutional scientific advisory committee of NIE, an independent committee for MAE-FETP, called the Board of Studies (BoS) and a team from SCTIMST periodically reviewed the performance of the programme. Specifically, the BoS reviewed the academic conduct of the programme (i.e., curriculum, teaching and evaluation methods).

### Logic model

We considered five key programme elements: (1) students, (2) curriculum, (3) faculty team, (4) field work and (5) laboratory support (Table 1). State public health departments nominated applicants who were accepted as students (input), who went through the programme (process), which resulted in graduates (output). For the curriculum, the identification of core competencies required for a field epidemiologist (input)and the curricular process for their acquisition (process) resulted in a documented, competency-based curriculum (output). For the faculty, institutionally affiliated staff (input) developed their skills according to the learning approach of the FETP (process), which resulted in a qualified, autonomous team (output). For the fieldwork, assignments (as per the mandate to use students to deliver public health service) (input) to conduct field projects (process) resulted in reports (output). For the laboratory, access to a network, resources, equipment and supplies (input) allowed the use of laboratories during field projects (process) that resulted in documentation of the field reports (output). All components combined to contribute to sustainable outcomes, including a qualified workforce, a network of graduates, an institutionalized training capacity and evidence-based public health decisions.

**Table 1:** Logical model for the Field Epidemiology Training Programme (FETP), National Institute of Epidemiology (NIE), Chennai, Tamil Nadu, India, 2001–2007

	**Scholars**	**Curriculum**	**Faculty**	**Field work**	**Laboratory**
	⇓	⇓	⇓	⇓	⇓
**Input**	**· Scholars nominated by institutions**	**· Institutional recognition**	**· Faculty with institutional affiliation**	**· Mandate to investigate and/or access data**	**· Access to laboratory network**
		**· Core competencies**		**· Field assignment of scholars**	**· Laboratory resources**
					**· Equipment and supplies**
	⇓	⇓	⇓	⇓	⇓
**Process**	**· Selection process**	**· Curricular process**	**· Faculty development**	**· Learning-through-service field epidemiology projects**	**· Standardized procedures for laboratory confirmation**
	**· Acquisition of competencies**				
	⇓	⇓	⇓	⇓	⇓
**Output**	**· Graduates**	**· Documented, competency-based curriculum**	**· Qualified, experienced, autonomous team**	**· Disseminated field project reports**	**· Laboratory-documented investigations**
	**· Candidates for faculty positions**				
	⇓	⇓	⇓	⇓	⇓
**Outcome**	**· Workforce qualified in applied epidemiology**				
	**· Network of graduates**				
	**· Institutional training capacity**				
	**· Evidence-based public health decisions**				
	**· Self-sufficiency / sustainability of the programme**				

### Programme evaluation

#### Students

**Figure 1:**
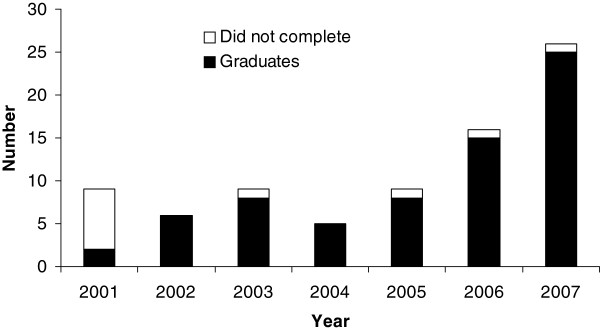
Students and graduates of the Field Epidemiology Training Programme (FETP), National Institute of Epidemiology (NIE), Chennai, Tamil Nadu, India, 2001–2007.

The number of eligible applicants increased from 9 in the 2001 cohort to 25 in the 2007 cohort (Table 2, Figure 1). Overall, 79 government nominees (public health service (75/79, 94%) and ICMR (4/79, 5%)) entered the programme between 2001 and 2007 (Figure 1). One student was a private applicant. The students were concentrated in West Bengal (24/79, 30%), Orissa (9/79, 11%) and Himachal Pradesh (9/79, 11%) where the programme became popular among public health officials. Of the 80 students who started the programme, 69 (86%) completed it and graduated (Figure 1). Of these, 55 (80%) responded to the survey, with a few skipping some items. The proportion of students who rated learning activities as “very useful” or better was 53/55 (96%) for practical exercises, 52/55 (94%) for field work, 51/55 (93%) for case studies, 50/55 (91%) for scientific conferences, 37/43 (86%) for field exercises, 39/46 (85%) for mini-contact sessions, 45/55 (82%) for classroom teaching and 45/55 (82%) for one-to-one student-mentor sessions. However, free-format comments suggested that training for the use of statistical software was insufficient.

**Table 2:** Evaluation indicators for the Field Epidemiology Training Programme (FETP), National Institute of Epidemiology (NIE), Chennai, Tamil Nadu, India, 2001-2007

	**Programme components**				
	**Students**	**Curriculum**	**Faculty**	**Field work**	**Laboratory**
Input	▪ 86 applicants, of which 79 (92%) nominated by states	▪ Course recognized by Medical Council of India (MCI)	▪ Faculty with ICMR staff status	▪ Posting within the public health service	▪ Institutional links with ICMR and other Government laboratories
		▪ 7 FETP-compatible core competencies^a^	▪ Faculty -to- student ratio ranging from 0.40 to 0.12	▪ 75% of the time spent practicing in the field	▪ Laboratory resources
					▪ Equipment and supplies
Process	▪ All state-nominated candidates selected^b^	▪ Curricular process documented and available on-line	▪ 1.3 supervision visits and 1.9 mini contact session/FETP course	▪ 76% to 100% fieldwork assignments rated at least “very useful” by graduates	▪ 66% graduates evaluating the laboratory component as insufficient
	▪ 82% to 96% of learning activities rated “very useful” or better by graduates				▪ 63% graduates rating laboratory curriculum “fair” or less
Output	▪ 69 graduates out of 80 students (86%)	▪ > 50% of graduates at least “proficient” for all competencies^c^	▪ >50% of graduates rating supervisions as “good” or better	▪ 47 oral presentations	▪ 76/106 (72%) investigations with laboratory-confirmation
				▪ 110 posters	
				▪ 29 publications	

^a^Revised in 2004.

^b^Selection process based on written examination and on personal interview implemented since 2009 in view of the large number of applicants (50% of applicants selected).

^c^Self assessment during the on-line survey. However, all students had to demonstrate acquisition of the competency in order to graduate.

### Curriculum

The Medical Council of India (MCI) recognized the MAE degree. However, the state public health care system had not formulated directives to use this degree as an official criterion for career promotion. The programme had seven core competencies: (1) mastering epidemiological sciences, (2) managing surveillance, (3) investigating outbreaks, (4) operational research, (5) human subjects’ protection, (6) communication and (7) programme management and evaluation. The curriculum was fully documented and from 2006, all tools were available on-line. The BoS and SCTIMST reviewed the curriculum every three years. The results of the exit survey suggested that most graduates self-assessed themselves as proficient (i.e., experienced in the field to the point that one can implement without supervision) for all core competencies at the end of the programme (Table 3). Most graduates had started the programme being only aware of six of the seven competencies. However, most graduates reported that they had started the programme without any competency at all in operational research (Table 3).

**Table 3:** **Self-assessed competencies among Field Epidemiology Training Programme (FETP) graduates, National Institute of Epidemiology (NIE), Chennai, Tamil Nadu, India, 2001–2007**^a^

	**Self assessed competency level**								**Gradient**
	**Before the programme**				**Upon completion of the programme**				
	**Mode/median**	**# with this answer**	**Total answers**	**%**	**Mode/median**	**# with this answer**	**Total answers**	**%**	
1. Epidemiology	Aware	34	58	59	Proficient	28	58	48	+2
2. Surveillance	Aware	30	58	52	Proficient	23	58	40	+2
3. Outbreaks	Aware	31	58	53	Proficient	28	58	48	+2
4. Research	None	30	58	52	Proficient	30	58	52	+3
5. Ethics	Aware	26	58	45	Proficient	27	58	47	+2
6. Communication	Aware	24	58	41	Proficient	33	58	57	+2
7. Programmes	Aware	26	58	45	Proficient	32	58	55	+2

^a^Scale included in the May 2009 survey: 0 = None, 1 = Aware (Has been exposed), 2 = Acquainted (Has experience but could not implement), 3 = Proficient (Can implement without supervision) and 4 = Expert (Can implement to the point s/he could supervise others).

### Faculty

Core members of the faculty were ICMR staff (Table 2). The faculty-to-student ratio ranged between 0.4 (2002) and 0.12 per student (2007, expected standard: 0.25). In addition, a WHO technical adviser was present full-time for two years from 2004, half time for two years from 2006 and 10% of the time for one year from 2008. Graduates reported having been exposed to an average of 1.9 supervision visits and 1.3 mini-contact sessions in the field during their three field postings (against a theoretical expected total number of three). When asked to rate the supervision in its various forms according to time allocated, feedback quality, conviviality, usefulness and encouragements, the majority of students (mode and median) rated supervision as good (Second best behind exceptional on a scale of four) for all the criteria (Data not shown).

### Field work

**Figure 2:**
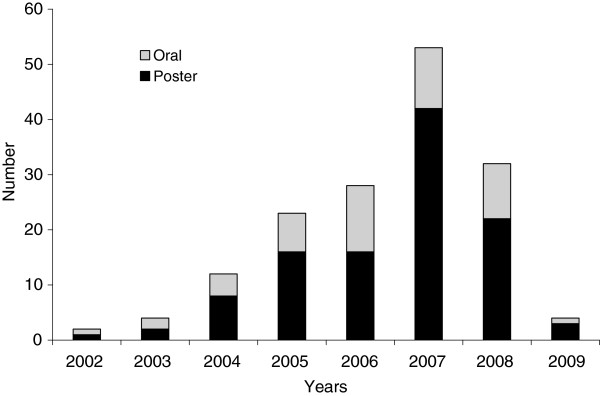
Scientific production of the Field Epidemiology Training Programme (FETP), National Institute of Epidemiology (NIE), Chennai, Tamil Nadu, India, 2001–2009.

All students but one had the mandate to deliver public health service through field practice. The remaining student worked mostly as a clinical epidemiologist in a hospital. Students spent 75% of their time in the field, as per plans. All 69 graduates completed their required deliverables, as this was a graduation requirement. Forty-two (78%) of 54 graduates from whom the information was available reported that the duration of the fieldwork was “just right”. The proportion of the graduates who reported that the assignment was “very useful” or “essential” was 54/54 (100%) for outbreak investigations, 52/54 (96%) for the dissertation, 52/54 (96%) for the programme evaluation, 51/54 (94%) for the surveillance data analysis, 51/54 (91%) for the surveillance evaluation and 41/54 (76%) for the situation analysis. There were 158 presentations at international meetings (110 posters and 48 oral presentations, a ratio of 2.3 per graduate). Topics of these presentations included outbreak investigations (n = 70, 44%), operational research and programme evaluation in infectious diseases (n = 34, 21%), surveillance (n = 15, 10%) and others, including non-communicable diseases, nutrition and injuries (n = 39, 25%). In addition, 29 scientific papers were accepted for publication in peer-reviewed journals (a ratio of 0.4 per graduate). There was an increase of scientific output as the programme enrolled more students (Figure 2).

### Laboratory

ICMR had its laboratory network, which allowed the sending of specimens to reference institutes. In addition, students developed links within their states, according to past collaboration histories. Laboratories covered the costs of testing. From 2005 onward, students also learned to work with the laboratory through a special laboratory-for-epidemiology module that was developed in collaboration with WHO headquarters and pilot tested in India. Twenty-four of 38 graduates (63%) from whom the information was available thought the laboratory component of the curriculum was relevant to their future career plans. However, 27/41 graduates (66%) assessed its quantity as “rather short” or “insufficient” and 26/41 (63%) assessed its quality as “fair” or less. Overall, of 106 outbreaks investigated, 91 (85%) had some form of laboratory documentation while 76 (72%) had a laboratory diagnosis (Table 2), without substantial variation over time (Data not shown).

### Outcome

All the programme graduates worked in state health departments in India. In 2009, six held leadership positions at the state level while the majority were working as programme managers at the district/sub-district level [14]. The qualitative component of the survey reflected this as most graduates considered that the FETP had “somewhat advanced their career”. Since 2008, a formal network of graduates led to activities, including an e-mail forum. The development of the institutional training capacity in the context of the ICMR school of Public Health led NIE to also start a Master of Public Health in 2008. As of 2009, publication of FETP investigations of outbreaks of cholera [15], typhoid [16,17], malaria [18,19], hepatitis E [20] and measles [21,22] provided shareable evidence of the use of epidemiological data to make public health decisions [3]. While WHO funded the first two cohorts, the Government of India fully funded the programme from 2003 onwards to ensure sustainability. WHO, however, provided a technical adviser during 2004–2009.

## Discussion

During 2001–2007, the programme established itself and grew, using standard competencies [3]. If the second cohort is left aside (start-up years faced initial difficulties), the number of graduates increased from 9 to 26 between 2003 and 2007 (+288% in five years). In states such as West Bengal, Orissa and Himachal Pradesh, the graduates played a crucial role, advocating with state governments for them to nominate candidates. The programme continued to achieve its objectives despite the increase in students. In addition to the acquisition of competencies, public health service was delivered through the production of information for action, some of which was reflected in conference presentations and publications. An excessive focus on such products could send the wrong message to public health managers who might perceive the programme as excessively academic. However, the constant increase in scientific production indicated that the quality was compatible with international standards. Moreover, as per prior studies, such products represent a useful indicator of future career success [9]. Senior State level health managers acknowledged the contribution of the students towards improvements in health services either to the faculty member during the field supervision (Himachal Pradesh, Orissa) or at national level meetings (West Bengal) [22]. Finally, the Indian public health system managed to retain this trained workforce in field positions even after graduation. This is similar to the experience of the EIS programme, wherein EIS officers assigned to the field had a tendency to stay in the field after graduation [8,9].

Aside from strengths, we identified areas for improvement. One weakness was the faculty-to-student ratio that dropped under the level of 0.25 per student initially pledged. While this did not affect the programme quality according to the indicators examined, it placed the faculty under a pressure that could have longer-term adverse effects. Conscious of this issue, the management decreased the number of admissions from 26 in 2007 to 15 from 2008 onwards. In 2009, the management also implemented a new system to screen state-nominated applicants through a selection process. This will (1) restrict admissions to the best applicants and (2) advocate for the quality of the programme. This, in turn, may generate higher quality applications. Laboratory support was another area identified for improvement. Institutionalization of the collaboration between epidemiologists and laboratory scientists is key to a functional disease prevention system [1]. From 2005 onwards, an increased utilization of the laboratory network of the Indian Integrated Disease Surveillance Project (IDSP) provided additional opportunities of laboratory confirmations. Finally, the use of computer programs to analyze data deserved more attention. This component of the curriculum faced a number of challenges, including (1) the heterogeneity of the target audience in terms of baseline computer skills, (2) the need for computers and statistical software and (3) the need for substantial faculty/tutor time.

Our evaluation suffered from three main limitations. First, for the purpose of this evaluation, the students self-assessed their competencies in the on-line survey. This could have over-estimated the competency level actually achieved. Second, this evaluation was only internal. Hence, we may have been subjective. To address this issue, we selected indicators that were as objective as possible and used a generic framework proposed by the United States CDC for FETPs. We also added a survey of graduates. Overall, we may have overlooked some of our limitations or generated an overly optimistic picture. However, our internal evaluation allowed us to identify areas that need improvement, which was our main objective. Third, our evaluation mainly focussed on input, process and output indicators of the programme. It did not address longer term outcomes and impact such as changes brought to the health system. These aspects will require a longer term perspective, maybe in the context of an external evaluation that our programme is willing to conduct in the future.

The establishment of a first FETP in India constitutes a proof of concept documenting that this approach was possible. Key innovations that this FETP brought to India included (1) the learning-through-service principle by which students in training deliver public health service and (2) the use of field epidemiology methods to investigate outbreaks using a sequence of descriptive and analytical epidemiology. Future work will need to focus on continuing investments in human resources to ensure a sufficient faculty-to-student ratio and additional curriculum innovations in the area of the laboratory and the use of statistical software. Most importantly, the FETP needs to be scaled up as the current number of graduates barely reaches about 10% of all the districts of the country that need a qualified epidemiologist. To provisionally address this gap, a shorter, two-week course was developed in 2008 to build basic epidemiological capacity in a lower-tier of professionals. This approach was also used in Germany [7] and Central America [23]. The National Centre for Disease Control (formerly National Institute of Communicable Disease) in Delhi scaled up this two-week course to train district epidemiologists in the country and also started a Master of Public Health in Field Epidemiology in 2006. However, to reach a critical mass of field epidemiologists and scale up the various initiatives successfully into a full-dimension national FETP, additional organizations and institutions must participate, possibly involving MD (Community Medicine) programmes that have always been a reference in terms of public health training in India. Such partnership will require quality assurance and possibly, an accreditation mechanism. In parallel to scaling up efforts, additional work is needed so that the competencies identified, developed and taught in the context of the FETP can become professional competencies recognized and sought in the public health workforce in India [24].

## Competing interests

The authors declare that they have no competing interests.

## Authors’ contributions

TB, YH, PK, PM, MM, VR and RR conceived the study. YH, TB and RR designed the survey questionnaire. PM and RR analyzed the data. YH, PM, MM and VR drafted the manuscript. MDG and VK revised it critically. All authors read and approved the final manuscript.

## Funding sources

The FETP India is funded by the Government of India, including the Indian Council of Medical Research (ICMR) and the Government of the various participating states. The WHO technical adviser to the FETP was funded by the United States Centers for Disease Control and Prevention (CDC). TEPHINET and WHO provided financial assistance to support the travel of FETP students to international conferences.
